# Pharmacological Effects of Cisplatin Combination with Natural Products in Cancer Chemotherapy

**DOI:** 10.3390/ijms23031532

**Published:** 2022-01-28

**Authors:** Shaloam Dasari, Sylvianne Njiki, Ariane Mbemi, Clement G. Yedjou, Paul B. Tchounwou

**Affiliations:** 1 Environmental Toxicology Research Laboratory, NIH-RCMI Center for Health Disparities Research, Jackson State University, Jackson, MS 39217, USA; shaloam.r.dasari@jsums.edu (S.D.); sylvianne.njiki@students.jsums.edu (S.N.); ariane.t.mbemi@jsums.edu (A.M.); 2 Department of Biological Sciences, College of Science and Technology, Florida Agricultural and Mechanical University, 1610 S. Martin Luther King Blvd, Tallahassee, FL 32307, USA; clement.yedjou@famu.edu

**Keywords:** cisplatin, natural products, combination therapy, modes of action, cancer treatment

## Abstract

Cisplatin and other platinum-based drugs, such as carboplatin, ormaplatin, and oxaliplatin, have been widely used to treat a multitude of human cancers. However, a considerable proportion of patients often relapse due to drug resistance and/or toxicity to multiple organs including the liver, kidneys, gastrointestinal tract, and the cardiovascular, hematologic, and nervous systems. In this study, we sought to provide a comprehensive review of the current state of the science highlighting the use of cisplatin in cancer therapy, with a special emphasis on its molecular mechanisms of action, and treatment modalities including the combination therapy with natural products. Hence, we searched the literature using various scientific databases., such as MEDLINE, PubMed, Google Scholar, and relevant sources, to collect and review relevant publications on cisplatin, natural products, combination therapy, uses in cancer treatment, modes of action, and therapeutic strategies. Our search results revealed that new strategic approaches for cancer treatment, including the combination therapy of cisplatin and natural products, have been evaluated with some degree of success. Scientific evidence from both in vitro and in vivo studies demonstrates that many medicinal plants contain bioactive compounds that are promising candidates for the treatment of human diseases, and therefore represent an excellent source for drug discovery. In preclinical studies, it has been demonstrated that natural products not only enhance the therapeutic activity of cisplatin but also attenuate its chemotherapy-induced toxicity. Many experimental studies have also reported that natural products exert their therapeutic action by triggering apoptosis through modulation of mitogen-activated protein kinase (MAPK) and p53 signal transduction pathways and enhancement of cisplatin chemosensitivity. Furthermore, natural products protect against cisplatin-induced organ toxicity by modulating several gene transcription factors and inducing cell death through apoptosis and/or necrosis. In addition, formulations of cisplatin with polymeric, lipid, inorganic, and carbon-based nano-drug delivery systems have been found to delay drug release, prolong half-life, and reduce systemic toxicity while other formulations, such as nanocapsules, nanogels, and hydrogels, have been reported to enhance cell penetration, target cancer cells, and inhibit tumor progression.

## 1. Introduction

In 2020, the burden of cancer in the U.S. was estimated to be 1.8 million new diagnosed cases of cancer, among which 43% accounted for prostate, lung, and colorectal cancer in men and 50% accounted for breast, lung, and colorectal cancer in women. The cancer mortality rate is higher among men than women and highest among African American men in terms of race/ethnicity [1].

Even though promising new treatment approaches have been implemented, cancer is still undefeated. Research has shown that certain risk factors are associated with the development of cancer, causing substantial morbidity and mortality rates. Some of the associated risk factors for the development of cancer include age, alcohol consumption, carcinogenic substances, chronic inflammation, diet, genetic mutations, hormones, immunosuppression, infectious agents, obesity, radiation, sunlight exposure, and tobacco usage. According to a new study from the American Cancer Society, approximately 42% of cancer cases and 45% of cancer deaths in the United States are associated with modifiable risk factors and therefore could be preventable [2].

A rise in the incidence rate is a reflection of a true increase in the disease occurrence and could also be attributed to new screening tests that result in a better diagnosis. So, the increase in cancer incidence does not change mortality rates. However, the best indicator of progress against cancer prognosis is comparison of the incidence and mortality trends, providing evidence for new improvised treatment methods. Statistical evidence suggests that improved treatments have probably made a significant contribution to the current sharp declines since the early 1990s [3]. The most recent annual report released in March 2020 showed that overall cancer death rates decreased by 1.8% per year among men and 1.4% per year among women (2001–2017) and among children aged 0–14 years (2013–2017) [4].

Cancer drug development has evolved from a low-budget government-assisted research aid to a high-stakes and multi-billion-dollar industry. With the advent of targeted therapy, the limitations associated with chemotherapy discovered by early researchers have been ruled out. Drugs are classified into two groups based on their toxicity: inhibitors of deoxyribonucleic acid (DNA) synthesis (e.g., cytosine arabinoside and methotrexate), and alkylating agents (e.g., Cisplatin, 5-fluorouracil, etc.).

Chemotherapy is one of the conventional modes of treatment for cancer malignancy. The first efforts were made by Goodman and Gilman in May 1942, from Yale School of Medicine, who observed a significant level of tumor regression after nitrogen mustard injection into the bloodstream of a patient with advanced non-Hodgkin’s lymphoma. The molecular action of the mustard compound, an alkylating intermediate (ethyleneimmonium ring) with high affinity towards electron-donating sites on proteins and nucleic acids, was attributed to the formation of a covalent bond with DNA and therefore the induction of apoptosis via alkylation of specific purine bases [5]. In the next two decades, cyclophosphamide, chlorambucil, etc., were administered to treat patients with lymphomas, leukemia, other solid tumors, etc. After the Second World War, Sydney Farber administered antifolates to children with acute lymphoblastic leukemia to suppress proliferation of malignant cells and restore normal bone marrow function. He observed that blocking the function of folate-requiring enzymes successfully induced remission. He received the credit as the first person to synthesize folate analogues (aminopterin and amethopterin) [6]. Methotrexate was the first solid tumor treated with drug therapy in humans with the help of Roy Hertz and Min Chiu Li at the National Cancer Institute [7]. Later, adjuvant chemotherapy was introduced in 1964 when Emil Frei and colleagues demonstrated the effect of methotrexate in combination with leucovorin for treating osteosarcoma [8]. George Hitchings and Gertrude Elion developed 6-mercaptopurine as an anticancer agent, which inhibits ribonucleic acid (RNA) and DNA synthesis [9]. This is a summary of the humble beginnings of modern chemotherapy. However, combinatorial treatment with a different site of action proved to be the most effective regimen against both metastatic cancer and in patients with a high risk of relapse after primary surgical treatment [10]. In addition, natural products were used for treating other comorbidities, such as diabetes, during the early 1950s due to the work of Eli Lilly’s natural products group on vinca alkaloids. The National Cancer Institute consists of a National Cancer Chemotherapy Service Center, established in 1951 for the development of animal models of cancer along with a range of transplantable solid tumors [11]. In 1990, researchers from Mayo Clinic found that the combination of 5-Fluorouracil with Levamisole was an effective treatment option for adjuvant therapy of colon carcinoma [12].

In this paper, we aim to provide a comprehensive review of the current state of the science highlighting the use of cisplatin in cancer therapy, with a special emphasis on its molecular mechanisms of action and treatment modalities including the combination therapy with natural products.

## 2. Research Approach

This study was carried out to review and discuss scientific information on the benefits and limitations of cisplatin use in cancer treatment, and to highlight the biomedical significance of natural products extracted from medicinal plants in addressing the challenges associated with cisplatin resistance and multiple organ toxicity.

We performed a search of MEDLINE, PubMed Central (https://www.ncbi.nlm.nih.gov/pmc/, accessed on 4 January 2022) database, Google Scholar, and other relevant sources to collect and review relevant publications on cisplatin, natural products, combination therapy, uses in cancer treatment, modes of action, and therapeutic strategies. A combination of key words and/or terms, such as cisplatin and cancer treatment, cisplatin mode of action, combination therapy of cisplatin and natural products, cancer prevention, and control, were searched. From the key term searches, we were able to identify peer-reviewed articles that reported invaluable information about the benefits and limitations of cisplatin use for cancer treatment, its mechanisms of action, and the strategic approaches being developed and applied to overcome cisplatin resistance and multiple organ toxicity, with a special emphasis on the combination therapy with natural products.

## 3. Cisplatin in Cancer Therapy

### 3.1. Structure and Synthesis

**Figure 1:**
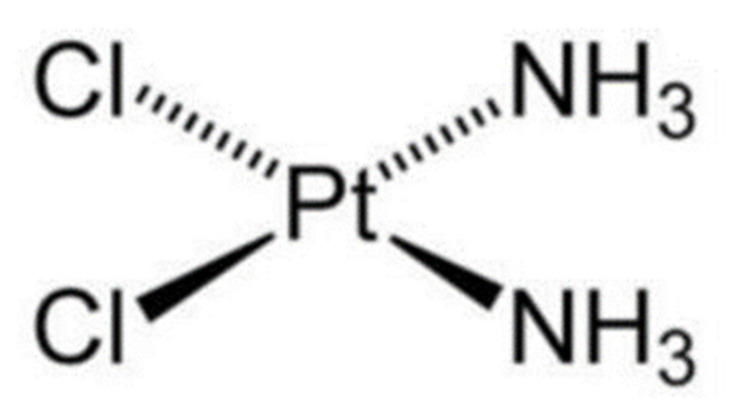
Chemical structure of cisplatin drug [18].

Cisplatin (PubChem CID: 5702198, MF-Cl_2_H_6_N_2_Pt; M. Wt-300.5; IUPAC name: dichloroplatinum) is a co-ordinate compound that has been widely used since its discovery in 1960 by Rosenberg at the University of Michigan [13]. In addition to testing its antibacterial activity against *Escherichia coli*, cisplatin’s anti-tumor activity in sarcoma and leukemia cell lines was evaluated in 1969 by the same group of researchers [14]. Using this preliminary data, clinical trials were conducted, introducing one of the most potent anticancer drugs to the clinic. In 1845, Michel Peyrone synthesized cisplatin for first time; later, Alfred Werner received the Nobel Prize award for disclosing the isomers of this platinum-based compound after identifying its structure (Figure 1) [15,16]. A rapid method for cisplatin synthesis was developed by Dhara in 1970, and later become a model for the majority of subsequent cisplatin syntheses [17].

### 3.2. Cisplatin Aquation

The replacement of Cl^−^ with a water molecule produces cis-[PtCl(NH_3_)_2_(H_2_O)]^+^, which is called aquation of cisplatin. Upon entry into the system, the chloride atoms on cisplatin are displaced by water molecules, forming an electrophile [19]. Hence, the hydrolyzed compound has an affinity to bind to the N7 of guanine on the DNA strand, forming 1, 2 intrastrand cross-links (chelation). This event forms kinked or bent DNA. However, DNA repair is blocked by newly bound proteins formed by binding to kinked DNA, forming high-mobility groups [20]. This cascade of signaling leads to induction of apoptosis. So, the anticancer properties of cisplatin arise from the substitution of the chlorine ligands with nucleophiles, such as DNA strand, hindering the replication of cancer cells. Interestingly, normal cells can manage DNA damage caused by cisplatin with the help of in-built repair mechanisms.

### 3.3. Mechanisms of Action

**Figure 2:**
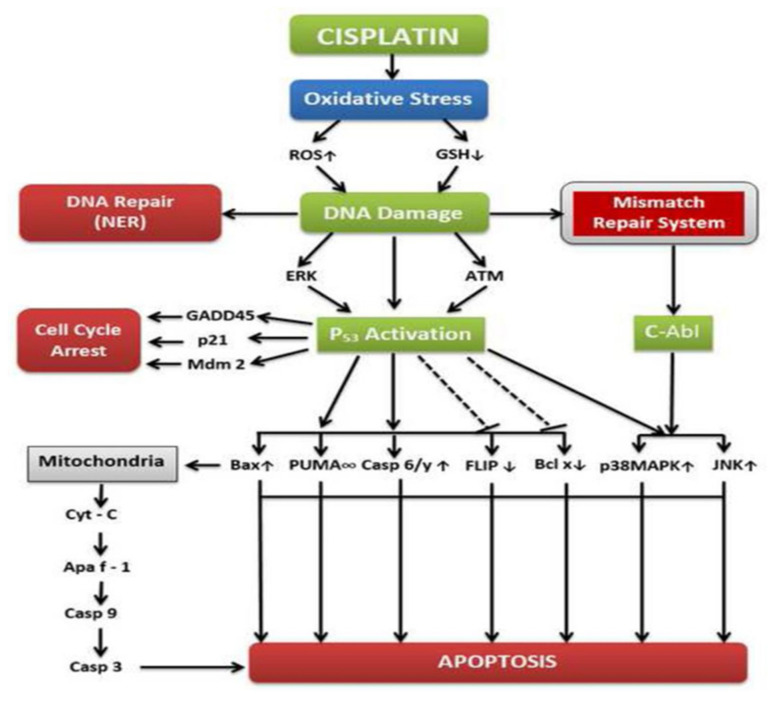
Overview of the molecular mechanisms of cisplatin in cancer treatment [21].

Cisplatin exerts its anti-tumor activity by covalent binding to DNA-forming adducts and therefore by triggering apoptosis [13]. Upon entry into the blood stream, cisplatin shows high affinity for sulfhydryl groups (proteins) and nitrogen donor atoms (nucleic acids), forming adducts due to aquation, which forms potent electrophiles [21]. The 1,2-intrastrand cross-links of purine bases with cisplatin account for 90% of the adduct formation, leading to its cytotoxicity. Even though DNA repair mechanisms are available, cells often undergo apoptotic or non-apoptotic cell death due to leftover impaired DNA, RNA, and proteins [22]. The mechanism of toxicity is a cascade of events starting with modulation of calcium signaling via copper transporters and therefore induction of oxidative stress [23]. This is followed by mitochondrial dysfunction and the leakage of vital membrane proteins that regulate caspases 8 and 9, thus resulting in activation of downstream or executioner caspases, such as caspases 3 and 7, and inducing apoptosis [24]. Hence, cisplatin interferes with signal transduction and cell regulation mechanisms, such as activation of ERK (extracellular signal-regulated kinase), phosphorylation of p53, upregulation of -p21, 45 kd-growth arrest and DNA damage (GADD45), mouse double minute 2 homolog (Mdm2), and phosphorylation of Bcl-2-associated death promoter (BAD) at ser136 via AKT, resulting in cell cycle arrest [25]. An overview of the molecular mechanisms of cisplatin toxicity is presented in Figure 2.

### 3.4. Clinical Studies on Cisplatin Use for Cancer Treatment

Approved by the United States Food and Drug Administration (FDA) in 1978, cisplatin has been widely used to treat several types of neoplasms: lung, ovarian, breast, bladder, testicular, and brain cancers, either alone or in combination with other drugs. However, the treatment dosage depends upon the stage of cancer and/or previous therapies received. Randomized phase II/III trails are being conducted on several projects related to cisplatin focused on areas of immunotherapy and radiation therapy among pediatric and adult patients. Certain analogues of cisplatin, such as carboplatin, ormaplatin, and oxaliplatin, are also under investigation in combination with other drugs to treat a variety of advanced metastatic cancers, causing tumor cells to shrink.

### 3.5. Cisplatin Resistance

Despite its potent action, cisplatin chemotherapy has some limitations associated with drug resistance and/or multiple organ toxicity. Several underlying factors, such as the uptake and efflux of the drug, overexpression of metallothionein, enhanced DNA damage repair system, nuclear respiratory factor 2 (Nrf2) signaling, epithelial-mesenchymal transition, and autophagy, contribute to cisplatin resistance, limiting its effective usage.

#### 3.5.1. Pharmacokinetics of Drug

An ideal drug distribution consists of effective drug uptake, maximum drug absorption, and distribution to the target organ. However, in case of treatment with cisplatin, reduced uptake of the drug and increased efflux has been observed, leading to drug resistance. As mentioned earlier, CTRs (copper transporter proteins) play a vital role in cancer resistance against cisplatin [26]. CTR1 is major protein responsible for cisplatin uptake [27]. In addition, overexpression of P-glycoprotein and MDR1 (multidrug resistance 1) leads to cisplatin resistance by effluxing the drug at a higher rate, resulting in less time to exert its bioactivity [28].

#### 3.5.2. Increased Affinity

Upon entering the blood stream, cisplatin becomes amphoteric and has high affinity for the sulfhydryl group in glutathione-S-transferase (GST) and metallothionein, forming a complex. This results in low bioavailability of cisplatin. Several studies have been conducted to investigate the role of metallothionein and GST in cisplatin resistance [29,30]. The translocation of metallothionein from the cytosol to the nucleus appears to protect DNA from damage, inhibiting the cytotoxic effect of cisplatin.

#### 3.5.3. DNA Repair and DNA Damage

Cells have innate DNA repair mechanisms (nuclear excision repair and mismatch repair protein); however, cisplatin treatment can cause cells to go through apoptosis due to impaired DNA. Interestingly, studies show that regulation of certain proteins, such as ERCC1 (excision repair cross-complementing-1), leads to cisplatin resistance. ERCC1 overexpression is a biomarker used for the prediction of cisplatin resistance because it is essential for NER of DNA, facilitating interstrand crosslink repair [31]. Hypermethylation of the MMR human homologue of MLH1 (hMLH1) accounts for 90% of cisplatin-resistant cell lines [32]. The resistance phenotype seems to be associated with enhanced NER and MMR repair systems, promoting cell proliferation. Another way of bypassing DNA lesions is through induction of translesion synthesis (TLS). Increased TLS has been noted in cisplatin-resistant cells due to overexpression of the human homolog of *Saccharomycescervisiae* 3 (REV3) [33]. In addition, mutations in BRCA1/2 lead to resistance since cisplatin adducts are recognized in the S-phase of the cell cycle and treated by the homologous recombinational repair system [34]. Altogether, cisplatin resistance is linked to an enhanced repair system established by regulation of the expression of proteins. Gene slicing or silencing suggests ways to regulate sensitivity to and toxicity of cisplatin.

#### 3.5.4. Other Control Mechanisms

Cisplatin resistance has been associated with a few other control mechanisms, such as the Nrf2 signaling pathway, epithelial-mesenchymal transition (EMT) process, and autophagy. Recent studies showed that Nrf2 modulated GSH levels in the cytosol, leading to drug resistance [35]. Cisplatin treatment induced EMT-mediated drug resistance as it stimulated macrophages to secrete chemokine ligand 20 (CCL20) to recruit t-helper cells to maintain an immunosuppressive tumor microenvironment, thus leading to cell proliferation [36,37]. In addition, scientific evidence exists demonstrating an induction of autophagy under hypoxia conditions linked to cisplatin treatment, causing a loss of sensitivity to therapy [38].

### 3.6. Organ Toxicity

Cisplatin has been known for its efficacy towards several types of cancers, such as germ cell tumors, sarcomas, carcinomas, and lymphomas. However, bioaccumulation of cisplatin has been noted, leading to multiple organ toxicity. In this section, we discuss th different types of cisplatin-induced toxicity along with current treatment options to alleviate the damage.

#### 3.6.1. Nephrotoxicity

Damage to kidneys, being the major organ in the human body for excretion, has been linked to cisplatin treatment due to both tubular secretion and glomerular filtration [39]. Drug efflux out of the body via the kidneys causes disproportionate retention of cisplatin at a rate of about five times that of the serum concentration compared to the liver [40,41]. The biotransformation of higher cisplatin thiols that trigger a glutathione imbalance is a suggested mechanism of its toxicity [42]. Histopathological studies have revealed proximal tubular damage as an early stage of toxicity, causing an imbalance in the reabsorption of water and sodium. In addition, more than 50% of the kidney tissue was disturbed after cisplatin treatment due to distal tubular damage, causing impaired renal flow of blood and glomerular filtration, which increases the secretion of proteins, enzymes, and electrolytes [43,44].

#### 3.6.2. Hepatotoxicity

The liver is an important organ that is responsible for several biochemical processes. An increase in the levels of malondialdehyde (MDA) and a decrease in antioxidant enzymes in the liver is an indication of liver toxicity to cisplatin treatment [45]. Elevated expression of cytochrome P450-2E1 enzyme leads to high levels of serum alanine transaminase (ALT) and aspartate aminotransferase (AST), and liver caspase-3 activity has been studied due to its cisplatin-induced hepatotoxicity [46]. Therefore, the oxidative stress mechanism could be a potential biomarker of the mitochondrial toxicity leading to liver injury among cisplatin-treated patients. However, several chemical agents have been documented for the prevention of cisplatin-induced hepatotoxicity, such as zinc, selenium, fosfomycin, sodium thiosulfate, *N*-acetyl-cysteine, methionine, and taurine [47].

#### 3.6.3. Neurotoxicity

Cisplatin induces toxicity in the nervous system depending upon the dose level and cumulative dose administered [48]. Studies have shown the presence of DNA adducts in peripheral nerves, causing peripheral neuropathy with clinical symptoms, such as automatic neuropathy, loss of haring, seizures, Lhermitte’s sign, and encephalopathy [49]. In addition, the dorsal root ganglion is considered the primary target of cisplatin-induced neurotoxicity due to its overproduction of reactive oxygen species (ROS) [50]. Few neurotropic factors, such as sulfur thiols, free oxygen radical scavengers, and phosphoric acid antibiotics, have been studied to prevent cisplatin-induced neurotoxicity [39].

#### 3.6.4. Cardiotoxicity

Cardiotoxicity is a well-known outcome of cancer chemotherapy among long-term cancer survivors after cisplatin treatment. The mechanism of damage includes a significant increase in lactate dehydrogenase, creatine kinase, creatine kinase isoenzyme MB, and plasma cardiac troponin I in the serum plasma concentration, followed by a substantial increase in the MDA level. In addition, significant decreases in the GSH content, SOD activity, and total protein content were observed in myocytes [51]. Clinical symptoms of heart damage induced by cisplatin treatment include cardiac ischemia (bradycardia), diastolic disturbances, hypertension, and microalbuminuria [52]. The use of specific nicotinamide adenine dinucleotide phosphate hydrogen (NADPH) oxidase inhibitors seems to have an ameliorative effect on cisplatin-induced cardiotoxicity [53].

#### 3.6.5. Other Organ Toxicity

**Figure 3:**
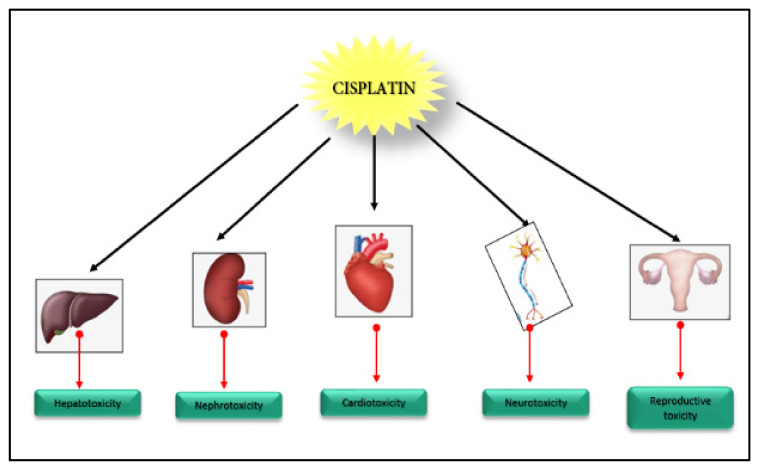
The current understanding of cisplatin chemotherapy-induced toxicity.

Ototoxicity has been mentioned in the literature as one of the side effects of cisplatin chemotherapy. Studies have shown that about 28% to 77% of patients show altered hearing thresholds after long-term exposure to cisplatin. The mechanism behind such damage appears to be overproduction of ROS in the cochlea, leading to apoptosis of the outer hair cells, spiral ganglion cells, and the stria vascularis [54]. Testicular toxicity is also induced by cisplatin chemotherapy as a long-term effect in few patients [55]. Figure 3 provides a schematic presentation of cisplatin-induced multiple-organ toxicity.

## 4. Natural Products in Cancer Management

Natural products (NPs) are chemicals secreted from secondary or non-essential metabolism by plants, microbes, other marine organisms. Despite the fact that 99% of organic compounds are synthetically prepared, more than one-third of all drug sales are based on natural products [56]. Recent studies have shown that over 120 different NP databases and collections have been published since 2000 [57]. Hence, it is apparent that NPs have increasingly attracted the interest of the scientific community as they are the source of most active ingredients of medicines. NPs have evolved over time and acquired a specific chemical diversity, with unique biological activities and drug-like properties. NPs have a significant history as active ingredients of traditional medicines (herbal remedies), from ancient Indian Ayurveda, traditional Chinese medicine, and African herbal medicines. About 2.1 million bioactive molecules with drug-like properties have been identified [58]. A huge variety of plant, microbe, and animal NPs; semi-synthetic NPs; and NP-derived compounds are under clinical investigations to treat infectious diseases, neurological conditions, cardiovascular and metabolic comorbidities, and immunological, inflammatory, and associated diseases [59]. NPs are cost effective and also protect against deleterious side effects unlike conventional chemotherapy. Nevertheless, a limited access and supply, the complexities of NP chemistry and feasibility of working with natural products, and ethical issues regarding intellectual property rights are some of the disadvantages of using NPs in drug discovery. In the following section, we will briefly discuss the few medicinal properties of NPs.

### 4.1. Garlic and Cancer Treatment

Plants of the *Allium* genus, such as garlic (*Allium sativum*), have played important dietary and medicinal roles throughout history. The medicinal potency of garlic has been widely used and known for over 5000 years. Almost all civilizations in the world had knowledge of the medicinal properties of garlic and used it for healing a variety of disorders, including leprosy, earaches, diarrhea, asthma, and constipation and to treat parasitic infection [60]. However, Weisbeger and Pensky demonstrated in the late 1950s the potent anticarcinogenic effects of garlic, reporting that thiosulfinates extract from garlic possessed antitumor properties [61]. According to the food program design of the U.S. National Cancer Institute initiated in the 1990s to determine which foods played an important role in cancer prevention, garlic may be the most potent food with cancer preventive properties [62].

The intake of garlic in a routine diet is linked to a reduced risk of cancer and has been recommended by the World Health organization (WHO), the National Cancer Institute, and the American Institute of Cancer Research (AICR) [63]. Epidemiological studies found a significant inverse relation between the consumption of 8.4 g or 33.4 g of raw garlic or garlic components a week for 7 years and lung cancer in a Chinese population [64]. Furthermore, the intake of garlic supplements <0.60 to > 3.65 kg per year for 2 years was significantly associated with a decreased risk of colorectal adenoma, which is a precursor of colorectal cancer [64,65]. The beneficial effects of garlic are attributed to its main bioactive agents, organosulfur compounds [66,67]. The organosulfur compounds that contribute to the anticancer properties of garlic include dially sulfide, dially disulfide, dially trisulfide, S-ally cysteine, allymercaptan, allymethyldisulfide, allymethyltrisulfide, allicin, allixin, and ajoene [68]. Several mechanisms have been proposed to explain the cancer preventative effects of garlic and its related sulfur compounds. These include inhibition of cell proliferation and tumor growth, modulation of enzyme activities, free radical scavenging, inhibition of mutagenesis, induction of DNA damage, and cell cycle arrest [69,70,71]. In vitro and in vivo studies have demonstrated that the growth rate of cancer cell is reduced by garlic OSCs (organosulfur compounds), with cell cycle arrest occurring mostly in the G2/M phase and stimulating the mitochondrial apoptotic pathway [72,73,74]. For instance, Wang and his colleagues reported that dially trisulfide inhibited the cell growth of basal cell carcinoma and human melanoma A375 cells by enhancing the level of intracellular ROS, causing DNA damage and inducing endoplasmic reticulum stress and mitochondria-mediated apoptosis. A recent study reported that dially disulfide suppresses FOXM1-mediated proliferation and invasion in osteosarcoma by upregulating miR-134 [75]. Furthermore, mono, di, and tri allyl sulfides and ajoene significantly promote cancer cell apoptosis, following by increased DNA fragmentation and intracellular free-calcium, downregulation of Bcl-2, and upregulation of p53 and Bax [76]. Western blot analysis of human pancreatic cancer cells with the wild-type p53 gene (capan-2) and normal pancreatic epithelial cells (H6C7) treated with diallyl sulfide indicated the expression of p21, p53, Bax, fas, and cyclin B1 while downregulating the expression of Bcl-2, Akt, and cyclin D1 protein levels in capan-2 cells compared to H6C7 cells [74].

### 4.2. Curcumin and Cancer Treatment

Curcumin is derived from *Curcuma longa* rhizomes and has been used for medical purposes to treat various ailments. Additionally known as diferloymethane, curcumin is viewed as a promising candidate, with preventive and therapeutic effects on various cancers [77,78]. Structurally, curcumin consists of 2 aromatic ring systems containing an o-methoxy phenolic group, connected by 7 carbon linkers consisting of an α,β-unsaturated β-diketone moiety with a molecular weight of 368.38 and C_21_H_2_O_6_ as a chemical formula, belonging to the class of natural polyphenolic compounds and the family of Zingiberaceae, a perennial herb [79,80]. It contains several macromolecules, including lipids, carbohydrates, proteins, and minerals; has a yellowish color present in the rhizomes; and has been used over several decades in food coloring, cosmetics, and traditional herbal medicine across Africa, India, and China for wound healing, skin infections, burns, bites, and acne [81]. Epidemiological findings reported that curcumin could inhibit cancer initiation, progression, and development and enhance cisplatin’s effect by affecting various signaling pathways and molecular targets implicated in cancer metastasis [82,83]. In addition, research demonstrated that the anti-cancer effects of curcumin alone or in combination with cisplatin interfered with the cell proliferation cycle, transcription and growth factors, inflammatory cytokines, protein kinases, and other oncogenic molecules [83,84,85].

A study evaluating the stemness characteristics of lung cancer cells co-treated with curcumin and cisplatin showed suppression of colony formation, cell proliferation activity, and downregulation of stem cell marker proteins. Another study investigating whether curcumin alone or combined with cisplatin could activate the Janus kinase/signal transducer and activator of transcription (JAK/STAT3) signaling pathways associated with cell growth and proliferation in papillary thyroid cancer (PTC) cell lines and derived stems cell noted that curcumin alone repressed PTC cell survival in a dose-dependent manner and modulated the gene expression STAT-3 associated with cell growth. The combined treatment of curcumin and cisplatin synergistically caused suppression of cell migration, activation, and phosphorylation STAT-3, and downregulation of stemness markers in thyroid cancer [86]. Similar research demonstrated that curcumin and cisplatin co-treatment increased bladder cancer’s apoptosis rate compared to cells exposed to single-agent treatment conditions. They also found that the co-treated bladder and cancer cells caused an increase in p53 and p21 gene expression and decreased STAT3 transcription activator, ROS production, and caspase-3 activation compared to the control [87,88,89]. Previous in vivo and in vitro studies using head and neck cancer cells found that combined treatment of cisplatin and curcumin inhibited tumor angiogenesis by 62%, VEGF production by 83%, STAT3 phosphorylation by 94%, and CAL27-CisR tumor growth by 77% compared to the non-treated group [90].

Saghatelyan and his co-investigators, using a randomized double-blind placebo-controlled clinical study in which patients intravenously received a 300 mg solution of curcumin along with 80 mg of paclitaxel once a week for 12 weeks, assessed the efficacy and safety of co-treatment of curcumin and paclitaxel among women with advanced and metastatic breast cancer [91]. After three months of follow-up, the results revealed that the treated group of women showed reduced fatigue compared to the placebo group (OR = 3.7; *p* < 0.01). It was also noted that the treated group had a significantly higher overall physical performance in terms of quality of life, time of tumor progression, and progression-free survival compared to non-treated patients (OR = 2.64; *p* < 0.01) [91]. Another pilot clinical study in which prostate cancer patients were administered 3 g/day of curcumin for 20 weeks showed that the curcumin-treated group showed a radio-protective effect compared to the non-treated group when undergoing radiation therapy by decreasing the severity of radiotherapy-related urinary symptoms [92]. However, clinical trials involving phase I or phase II treatment of cancer patients with curcumin or combination data remain controversial and inconclusive in terms of complete cancer remission [93].

### 4.3. Ascorbic Acid (Vitamin C) and Cancer Treatment

Ascorbic acid is one of the first major defense systems against aqueous radicals in the blood and it plays a critical role in protecting biological membranes against primary peroxidative damage. It has the ability to reduce oxygen, nitrogen, and sulfur radicals at non-toxic doses [94]. Scientific reports have discussed the role of ascorbic acid in general medicine and anticancer therapy.

Ascorbic acid has been used in combination with cisplatin as an adjunctive treatment for many cancers [95,96,97,98]. It has a wide range of pharmacological effects including anticancer, anti-inflammatory, antioxidant, anti-diabetic, wound healing, and anti-osteoporotic effects. Kurbacher et al. demonstrated that ascorbic acid increased the cytotoxic activity of chemotherapeutic drugs including doxorubicin, cisplatin, and paclitaxel in breast cancer cell lines [99]. Longchar and Prasad showed that pre-treatment with ascorbic acid in combination chemotherapy ameliorated cisplatin-induced oxidative stress and decreased alterations in the antioxidant defense system and significantly decreased cisplatin-induced mutagenicity in hosts [95]. A similar study demonstrated that ascorbic acid decreased nephrotoxicity caused by cisplatin without a reduction in the efficacy of cisplatin in C57BL/6 mice with Lewis lung carcinoma [100]. Additional scientific reports have demonstrated that ascorbic acid and cisplatin co-treatment may protect kidney and normal cells against cisplatin-induced nephrotoxicity and DNA damage in vivo [101,102]. A previous investigation revealed that the therapeutic effect of cisplatin was more favorable when combined with ascorbic acid than its single use in tumor-bearing mice [103]. This investigation also revealed that the combination treatment of ascorbic acid plus cisplatin reduced tissue toxicity in the hosts with better antitumor activities [103]. Scientific data have indicated the role of ascorbic acid in cisplatin-induced nephrotoxicity and mutagenicity in animal models and humans [101,104]. Experimental studies have indicated that ascorbic acid or l-carnitine is very effective at preventing oxidative damage [101,105]. One research study indicated that ascorbic acid and l-carnitine treatment ameliorated cisplatin-induced nephrotoxicity in rats due to their antioxidants and anti-inflammatory properties [96]. This combination treatment suggests that ascorbic acid and l-carnitine may enhance the antioxidant defense mechanism and membrane stability of the renal cell.

### 4.4. Ginger and Cancer Treatment

*Zingiber officinale* (ginger) is an ancient spice that is normally used as a flavoring agent for food, and it is listed on the Food and Drug Administration (FDA) safe list [106,107]. Ginger and its isolated constituents, such as [6]-gingerol, [6]-paradol, phenolic 1,3-diketones, and zingerone, possess pharmacological properties including anti-bacterial, anti-cancer, anti-fungal, anti-inflammatory, anti-mutagenic, antioxidant, anti-viral, and immunomodulatory activities, that are associated with its several bioactive compounds, including volatile oil, polyphenols, and flavonoids [108,109,110]. A scientific report revealed the protective effects of ginger extracts against toxicities induced by toxicants [111]. Few studies have evaluated the therapeutic effects of ginger co-treatment with cisplatin. A recent study indicated that cisplatin-induced testicular alterations were considerably mitigated by fresh ginger juice through abrogation of oxidative stress and an anti-inflammatory mechanism, providing evidence for the antioxidant and anti-inflammatory effects of ginger juice on cisplatin testicular damage [112]. Several studies demonstrated that ginger exerts its antioxidant activity through its active antioxidant ingredients, such as zingerone, zingiberene, gingerdiol, gingerrols, and shogaols, which improve oxidative stress by decreasing lipid peroxidation reaction and free radical scavenging, protecting DNA, and enhancing the activity of antioxidant enzymes [113,114]. Cisplatin is largely used in the treatment of many cancers, including bladder, cervical and endometrial tumors, head and neck, lung, lymphoma, and sarcoma [115,116]. However, its use has been limited due to its harmful effects on the kidneys, peripheral nerves, inner ear, and testicles [117,118]. Therefore, ginger co-treatment with cisplatin is attractive. A study indicated that zingerone, a constituent of ginger, provides protection against cisplatin-induced oxidative damage in the jejunum of Wistar rats [119]. The combination of ginger and sodium salicylate nanoemulsion showed a hepatoprotective effect on the amelioration of cisplatin-induced hepatotoxicity in an animal model [120].

### 4.5. Vernonia amagdalina (VA) and Cancer Treatment

VA is a valuable edible plant that is widespread in West Africa and it is well appreciated in Cameroon for its nutritional and medicinal properties [121,122]. It is commonly called bitter leaf in English because of its bitter taste, and it possesses significant medicinal and nutritional values. Herbalists and naturopathic doctors in West Africa recommend aqueous extracts of VA to patients for the treatment of human diseases, including cancers [121,122,123]. Several studies reported that VA possesses anticancer and antioxidant properties, which correlate with its medicinal properties. The anticancer and antioxidant activities of phytochemical extracts of VA have been established in different scientific studies [124,125,126]. Scientific studies demonstrated that phytochemical extracts of VA and other *Vernonia* species, including *Vernonia calvonia*, *Vernonia divaricate*, *Vernonia amygdalina* Delile, and *Vernonia guineensis* Benth, exhibit anticancer activities and are valuable sources for drug-like active natural compound screening [127,128,129,130]. The major identified phytochemicals of VA responsible for its ethnobotanical uses include flavonoids, alkaloids, saponins, terpenes, ligands, cournarins, phenolic acids, sesquiterpenes, and edotides [131,132,133,134]. Other phytochemicals or bioactive compounds present in VA include vernonioside A1, vernonioside B1, vernonioside B2, vernodalin, vernomygdin, vernolepin, and vernodalinol, steroids, tannins, glycosides, and terpenoids [135,136,137,138]. These phytochemicals present in VA are indispensable for the human body. The anticancer activities of VA have been studied in various cancer cell lines. We demonstrated in our research laboratory that *Vernonia amygdalina* inhibited the proliferation of breast cancer (MCF-7) cells [139], *Vernonia amygdalina Delile* inhibited the growth of prostate cancer (PC-3) cells [128], and *Vernonia calvonia* caused growth arrest of ovarian cancer (OVCAR-3) cells [127]. Cheng et al. demonstrated that VA induced anti-cancer effects in MCF-7 and MDA-MB-231 cells via caspase-dependent and p53-independent pathways [140]. A previous pharmacological study of Izebigie revealed that water-soluble VA leaf extracts potently retarded the proliferative effects of human breast cancer (MCF-7) cells in a dose-dependent manner [133].

## 5. Cisplatin Combination with Natural Products

Treatment with NPs has been shown to be a promising remedy for cisplatin-induced toxicity. Research has been conducted to elucidate the protective role of NPs against cisplatin-induced toxicity in non-target organs. NPs help with chemo-sensitization of cisplatin-resistant cells by either up- or downregulating specific signaling pathways triggering apoptosis. In the sections below, we will discuss the effects of natural products in cisplatin chemotherapy.

### 5.1. Combination with Flavonoids

Flavonoids, the largest group of phytonutrients, are a class of polyphenolic secondary metabolites. They are subgrouped as anthocyanidins, flavanols, flavones, flavonols, flavonones, and isoflavones. The consumption of flavonoids increases the longevity of life, helps in weight management, treats cardiovascular diseases and diabetes, and prevents cancer and treats certain neurodegenerative diseases. Such natural compounds from plant sources are powerful in sensitizing cisplatin-resistant phenotypes, thus synergizing cisplatin treatment. A recent study was conducted to show the effect of Oroxylin A in hypoxia-induced cisplatin resistance by inhibiting HIF-1α-mediated xeroderma pigmentosum group C (XPC) transcription [141]. The sensitization of certain cancer cells to cisplatin after flavonoid treatment has been studied in both ovarian and prostate cancer cells [142,143]. The mechanism of flavonoid’s action in potentiating cisplatin treatment is depletion of the cellular oxidative machinery, aiding mitochondrial dysfunction and thus leading to apoptosis [144]. A few more examples of flavonoids, such as artocarpanone, artocarpin, cycloartocarpin, and cyanomaclurin, extracted from *Artocarpus heterophyllus* heartwoods have a synergistic effect with cisplatin on non-small lung and breast cancers [145]. Recent studies have shown that quercetin prevented cisplatin nephrotoxicity by ameliorating tubular damage [146]. Isoquercetin and rutin, flavonoids from *Morus alba*, have been shown to enhance the therapeutic efficacy of cisplatin significantly compared to a single dose of cisplatin alone in treating gastric cancer [147]. Studies on 6-methoxyflavone (gamma-aminobutyric acid (GABA) modulator) treatment in rats showed a protective effect on cisplatin-induced neuropathic allodynia and hypoalgesia [148]. Studies also showed that flavonoids have a protective function against the nephrotoxicity induced by cisplatin treatment, for example, galangin from ginger, naringin, rutin, and hesperidin from grape and citrus fruits [149,150,151]. The molecular mechanism behind the protection against nephrotoxicity appears to be inactivation of p53, mitogen-activated protein kinase (MAPK), and protein kinase B (AKT) signaling cascades and therefore apoptosis is not triggered [152,153].

### 5.2. Combination with Saponins

Saponins, structurally triterpene glycosides, constitute a group of terpenoids used in soaps, medicines, fire extinguishers, and dietary supplements. Recent literature on the cytoprotective effect of saponins shows their growing interest in the scientific community. Saponins protect against cisplatin-induced nephrotoxicity, gastric toxicity, cardiotoxicity, and ototoxicity [154,155,156]. One of the mechanisms of cytoprotection is carried out by inhibiting the mitochondrial apoptotic pathway by increasing HIF-α protein [154]. A few examples of saponins with biological importance include ginsenoside RK1, panax notoginsen, panax quinquefolius, and paris saponin-II [156,157,158]. In addition, saponins collected from flowers, such as *Camellia sinensis*, appear to sensitize cisplatin-resistant cells by inducing apoptosis in certain ovarian cancers [159,160]. A synergistic effect of saponins, such as β-elemene (*Rhizoma zedoariae*) and from medicago species, has been observed in cervical and breast cancer when treated in combination with cisplatin [161,162]. The effectiveness and sensitivity was shown to improve due to the multidirectional effects of saponin fraction isolated from plant sources in cisplatin chemotherapy.

### 5.3. Combination with Alkaloids

Alkaloids are colorless, crystalline, and non-volatile secondary metabolites with potent pharmacological effects. Alkaloids are predominantly synthesized by plants and few animal species (beaver and poison dart frogs) and other fungi (ergot). Studies show that the notable antiproliferative activity of certain alkaloids (zanthoaustrones) is equivalent to cisplatin when used against some human cancers [163]. In the past five years, alkaloids have been studied to increase the sensitivity to cisplatin therapy, especially in ovarian cancers [164,165,166]. Signaling pathways that enhance cell death include modulation of AKT-Kβ, c-Jun *N*-terminal kinase (JNK) to trigger cell cycle arrest, caspase activation, and eventually induction of apoptosis [167]. In addition, a significant amount of research has investigated the use of alkaloids to protect non-target organs, such as the kidney, liver, and nervous system, from cisplatin damage by blocking some pathways [168,169].

### 5.4. Combination with Polysaccharides

Natural polysaccharides are the main bioactive compounds in natural medicine due to their immunomodulatory, antitumor, liver protection, and anti-inflammatory properties [170]. The polysaccharides have gained much importance in the scientific community, especially in conjunction with cisplatin. For the past few years, Astragalus, a Chinese herb, has been used in combination with cisplatin to treat cisplatin-induced damage (nephrotoxicity) in a variety of cancers [171]. In addition, the same chemical compound seems to have a synergistic effect when combined with cisplatin to treat female reproductive cancers [172,173]. Hence, polysaccharides protect vital organs from toxicity and also sensitize cells to cisplatin treatment by altering autophagy or triggering apoptosis [173].

### 5.5. Combination with Phenylpropanoids

Due to their low toxicity and variety of bio-active properties, phenylpropanoids are widely tested in drug development. Chemically, phenylpropanoids are either plant based or synthetically derived from phenolics, a major subgroup of plant metabolites [174]. Commonly available phenylpropanoids include p-coumaric acid, caffeic acid, ferulic acid, and sinapic acid [175]. Recent studies have shown that phenylpropanoids are capable of reversing the damage caused by cisplatin treatment [176]. Acute liver and kidney damage induced by cisplatin treatment has been attenuated by using certain phenylpropanoids via improvement of antioxidant and oxidative stress parameters, including MDA [177]. In addition, phenylpropanoids synthesized in the laboratory, or isolated from NPs, such as Cirsum Japonicum and Alpinia officinarum rhizome extract, are reported to have a synergistic effect with cisplatin treatment [178,179].

### 5.6. Combination with Napthoquinones

Naphthoquinones are phenolic compounds synthesized from bacteria and fungi and plants during secondary metabolism. They have beneficial antibacterial, antifungal, antiviral, insecticidal, anti-inflammatory, and antipyretic properties. Synthesis, characterization, protein binding, DNA binding, and cytotoxicity studies on naphthoquinones are currently in high demand. Naphthoquinones have recently been discovered to have therapeutic efficacy as anticancer agents [180]. Not only do they exhibit cytotoxicity in certain cancer cells, but they also act as potential antineoplastic agents when combined with either cisplatin or its analogues [181,182]. The cytotoxic potential of naphthoquinones, such as lapachol and β-lapachone, is about 11 times more than cisplatin alone [183]. The molecular mechanism of their cytotoxicity is via induction of mitochondria-mediated apoptosis and cell cycle arrest [184,185]. In addition, certain naphthoquinones have been documented to reduce the toxicity exerted by cisplatin treatment [186]. Many derivatives of naphthoquinones have yet to be checked for their protective and antineoplastic role in cisplatin chemotherapy.

### 5.7. Combination with HSP90 Inhibitors

Heat shock proteins (HSPs) are novel targets for cancer therapy due to their protective role, allowing cells to live in extraneous stress conditions. HSP 90 and HSP 70 are common molecular targets in cancer therapy. Hence, HSP inhibitors work effectively by depleting key apoptotic proteins and thereby exhibit synergism with cisplatin treatment [187]. Several natural HSP90 inhibitors have been identified to have a potential role in overcoming cisplatin resistance [188]. Interestingly, among all natural HSP90 inhibitors, about one-third of new entities have been approved by the FDA [189]. 17-allyl amino geldanamycin (17-AAG) is the first clinically approved HSP90 analogue inhibitor to be isolated from *Streptomyces hygroscopicus* [190]. Naturally available HSP90 inhibitors are classified into groups depending on the binding location (Table 1).

### 5.8. Combination with Vitamin C (Ascorbic Acid)

Vitamin C is an important dietary supplement responsible for tissue repair and enzymatic production of neurotransmitters along with other functions. The best sources of natural vitamin C are citrus foods along with some other vegetables and fruits. Studies have been conducted on vitamin C and cisplatin since the late 1900s. Vitamin C protects vital organs, such as the kidney, male reproductive organs, etc., from cisplatin damage [104,191]. A recent study in bone marrow cells revealed that micro nucleated polychromatic erythrocytes induced by cisplatin were reduced with the use of antioxidants, such as vitamin C [192]. In addition, vitamin C in combination with cisplatin induces cell death by altering oxidative biomarkers in the cell and p53 upregulation, triggering apoptosis [193]. Such synergistic effects of vitamin c with cisplatin on the inhibition of the proliferation of cancer cells in a single dose have evolved into chitosan-embedded vitamin C nanoparticles against cisplatin-induced reproductive toxicity [194].

### 5.9. Combination with Other Natural Products

Several other NPs have been tested against cisplatin-induced toxicity and chemosensitizing cisplatin resistance cells. Resveratrol in combination with cisplatin has a synergistic effect in the treatment of lung cancer by modulating autophagy [195]. Triterpenoids, such as curcubitacin B, act as chemosensitizers, killing cisplatin-resistant cells by inducing apoptosis in ovarian cancer [196]. At the same time, terpenoids are also protective agents, such as Xanthorrhizol extracted from rhizomes of curcuma species, against nephrotoxicity induced by cisplatin [197]. Bee honey and royal jelly protect kidneys from subchronic cisplatin-induced renal injury by reducing the expression of α-smooth muscle actin and decreasing interstitial fibrosis [198]. Quinones, such as celastrol, extracted from the roots of *Tripterygium wilfordii,* can cause apoptosis though the extracellular signal-regulated kinase 1/2 (ERK1/2) and p38 MAPK signaling pathways in cisplatin-resistant cells [199]. Polyphenols are potent antitumor agents that have a role as natural products in cisplatin-induced damage. For example, pro-anthocyanidin from bayberry leaves inhibits the growth of new cells and regulates the cell cycle in cisplatin-resistant ovarian cancer cells via targeting of the Akt pathway [200].

## 6. Natural Product–Cisplatin Nanoparticle Formulations

The development of nanoparticle formulations of cisplatin is a novel method used to fight against cancer. Even though natural products have been shown to sensitize cisplatin activity in several cancers, achieving adequate bioavailability has been challenging regarding its anticancer potential. Hence, nanoformulations have been developed to increase their therapeutic efficacy. The advantages of nanotechnology include a delayed release, prolonged half-life, and reduced systemic toxicity. Polymeric, lipid, inorganic, and carbon-based nano-drug delivery systems have been studied in combination with cisplatin. Other nanoparticle formulations include nanocapsules, nanogels, hydrogels, silk fibroin, caseins, and fucoidan with enhanced cell penetration, which target tumors and inhibit tumor progression. Table 2 presents the available nanoparticle formulations studied in combination with cisplatin categorized by the type of nanodrug used.

### 6.1. Clinical Trials

Currently, some nanodrug formulations of cisplatin with NPs are under clinical investigations. Lipoplatin, a liposome-based platinum formulation, is undergoing phase III clinical trials for FDA approval [201]. Phase II clinical investigations on SPI-77 from ALZA Pharmaceuticals are in progress for the treatment of non-small carcinoma of lung cancer [202]. Nanoplatin is being investigated under phase III clinical trials with reduced nephrotoxicity [203]. Aroplatin^TM^ has cleared phase II clinical studies to be used for treating colorectal cancer [204].

### 6.2. Mechanism of Action

The therapeutic efficacy of nano-formulation displayed excellent results against cisplatin chemotherapy resistance. The ionic gelation method of curcumin-loaded chitosan nanoparticles inhibits the PI3k/AKT and Janus kinase (JAK)/signal transducer and activator of transcription 3 (STAT3) pathway and therefore potentiates the efficacy of cisplatin treatment [205]. The molecular mechanism that releases cisplatin from nanotubes with cancer-specific peptide sequences instead of conventional surfactant molecules showed slow release of the drug, causing effective therapy results [206]. Such a slow release mechanism of cisplatin from cubosomes appears to be pH dependent and encapsulating nanoparticles act as a physical barrier and enhance the controlled release of the drug at the target site [207]. Nano-formulations with different specific targets on the tumor linked with different metabolic pathways lead to induction of apoptosis [208]. Hence, polymeric-based nanoparticles, due to their nano-size and solubility, have a higher potential to cross the cell membrane and capillaries, allowing for a controlled release rate while maintaining the anti-cancer activity of cisplatin.

### 6.3. Other Natural Nanoparticle Formulations

Boldine is an aporphine class alkaloid, extracted from *Peumus boldus* (boldo). Boldine leaves have significant antioxidative properties and were traditionally used in folk medicine. Due to its medicinal properties, boldine has been extensively studied in recent years for the benefit of human health. The administration of nanoencapsulated boldine killed hepatocyte carcinoma while increasing the viability of normal liver cells treated with cisplatin [209]. Hence, boldine-loaded poly d-,l-lactic-co-glycolic acid (PLGA) nanoparticles not only improved drug carriage but were also protective against cisplatin-induced toxicity. Silk fibroin nanoparticles are also potent against cisplatin toxicity. According to recent literature, these polymers, produced from an insoluble protein present in silk produced by larvae of *Bombyx mori*, work as delivery vehicles to treat neuroblastoma [210]. Hence, silk fibroin NPs are in high demand due to their antitumor properties and controllable biodegradability [211]. Casein is a naturally occurring milk protein used as a vehicle to transport nanoparticles for enhanced targeted delivery. Because of their effective penetrating capacity beyond cell membrane barriers to target tumors, the growth inhibition of cisplatin-loaded casein nanoparticles is 1.5-fold higher than that of free cisplatin [212]. Fucoidan is another NP extracted from brown algae (*Fucus vesiculosus*, *Cladosiphon okamuranus*, *Laminaria japonica*, and *Undaria pinnatifida*). It has been studied for its use as a nanocarrier for effective delivery of cisplatin [213]. Their mechanism of action seems to be via upregulation of TLR4/CHOP-mediated caspase-3 and PARP activation, thus leading to cell death [214]. In addition, fucoidan alone is protective against cisplatin-induced renal damage and gastric issues [215,216].

## 7. Conclusions

Cisplatin is a potent chemotherapeutic drug used for the treatment of various human cancers. Its mode of action involves covalent binding to DNA, forming adducts and thereby triggering apoptosis and/or necrosis through a series of biochemical mechanisms that involve oxidative stress, DNA damage, and interference in various signal transduction pathways. However, its usage has been limited due to side effects and chemoresistance. After gaining an understanding of the molecular mechanisms underlying cisplatin resistance and multiple-organ toxicity, the scientific community is now searching for alternatives to enhance the bioactivity of cisplatin and to reduce or eliminate its side effects. Natural products have a wide range of properties that can be exploited for the benefits of human health. Not only do they have antibacterial, antifungal, antipyretic, and antiparasitic effects, but they also exert anticancer properties. Scientific evidence has demonstrated that a significant number of natural products have significant potential to protect against cisplatin-induced toxicity in multiple organs including the liver, kidneys, and cardiovascular, hematopoietic, reproductive, and nervous systems. Combination therapy of cisplatin and natural products has been shown to be effective in alleviating resistance to cisplatin treatment; underscoring the development and application of natural product-based formulations of cisplatin as a novel therapeutic strategy to fight human cancers.

## Figures and Tables

**Table 1:** Natural products acting as HSP90 inhibitors grouped depending on their binding affinity.

Binders	HSP90 Inhibitor	Source	Class
*N*-terminal domain binders	Ansamycins	*Streptomyces hygroscopicus*	Benzoquinone ansamycin
	Radicicol and pochonins	*Monosporium bonorden*	Macrocyclic lactone
	Geraniin	*Geranium thunbergii*	Tannin
	Gambogic acid	*Garcinia hanburyi*	Xanthonoid
	Panaxynol	*Panax ginseng*	Polyacetylene
	Deguelin	*Fabaceae (Leguminosae) plants*	Rotenoid
	Heteronemin	*Marine sponges*	Sesterpene
	C9-type iridoids	*Bignoniaceae plants*	C9-type iridoids
Middle-domain binders	Lentiginosine	*Astragalus lentiginosus*	Dihydroxyindolizidine alkaloid
	Kongensin A	*Croton kongensis*	Diterpene
	Sansalvamide	*Fusarium species*	Cyclic pentadepsipeptide
*C*-terminal domain binders	Derrubone	*Derris robusta*	Isoflavone
	Coumarin antibiotics	*Streptomyces niveus*	Aminocoumarin
	Epigallocathechin	*Camellia sinensis*	Catechins
	Fusicoccane diterpenes	*Alternaria brassicicola, Hypoestes forsskaolii*	Fusicoccane diterpene
Co-chaperone binders	Withaferin A	*Solanacea plants*	Steroidal lactones
	Cucurtabicin D	*Cucurbitacea plants*	Tetracyclic triterpenes
	Celastrol	*Tripterygium wilfordii; Celastrus regelii*	Triterpene
	Gedunin	*Meliaceae plants*	Triterpene

**Table 2:** Types and examples of cisplatin-based nanoparticle formulations for drug delivery.

Type of Nanodrug Delivery	Examples
Polymeric CDDP-based nanodrug delivery systems	Polylactic-co-glycolic acid nanoparticles
	Polyethylene glycol (PEG) nanoparticles
	Chitosan nanoparticles
	Micelles
	Dendrimers
	Polymer–drug conjugates
	Poly butyl cyanoacrylate (PBCA) based nanoparticles
	Poly aspartic acid (PAA) nanoparticles
	Polydopamine nanoparticles
	Glutathione-scavenging poly (disulfide amide) nanoparticles
	Albumin-based nano-formulations
	Gelatin NPs
Lipid-based nanocarriers for cisplatin	Liposomes
	Cubosomes
	Transfersomes
Inorganic nanoparticle-based nanodelivery systems	Gold nanoparticles
	Mesoporous silica nanoparticles
	Magnetic iron oxide NPs
	Calcium-based nanoparticles
	NaGdF4:Yb3þ/Er3þ nanoparticles
	Europium (III) doped yttrium vanadate nanoparticles
	Aluminum-doped MCM-41 nanoparticles
	Photothermal conversion nanoparticles
	Melanin nanoparticles
	Coordination polymer nanoparticles
Carbon-based nano-formulations for cisplatin	Carbon nanotubes
	Graphene
	Fullerene

## Data Availability

Not applicable.
